# Esophagotomy for the Removal of False Teeth1Read before a special meeting of the Clinical Society of the New York Polyclinic Medical School and Hospital.

**Published:** 1905-09

**Authors:** John A. Wyeth

**Affiliations:** New York


### Esophagotomy for the Removal of False Teeth.[1]

### With Some Remarks on Colon Alimentation.

JOHN A. WYETH, M. D., LL.D., New York.

[*From Journal of the American Medical Association.*]

HISTORY.—A man, 50 years old, a sculptor by occupation, in good health, on Monday, February 27, 1905, at 11 p. m., in a fit of laughter, dislodged and swallowed an artificial plate of vulcanised rubber to which two false upper incisor teeth were soldered.

This plate, crescentic in shape, measured from point to point along the arch two and one-half inches, the direct diameter between the two points of the crescent was one inch and a half, the widest measurement of the plate at the center, including the teeth, was five-eighths of an inch.

Two hours after the accident he was carried to the city hospital in Cincinnati. The house surgeon introduced his finger deeply into the pharynx, but did not feel any foreign substance. On account of severe spasmodic pains, the patient was given several hypodermics of morphia during that night. At 8.30 the next morning deglutition was extremely difficult, the patient complaining of severe pains in the throat and both sides of the chest.

At 11 a. m. of the 28th he was given a small piece of bread which, after thorough mastication and moistening with a draught of water, was swallowed. The surgeon who then visited him introduced a bulbous bougie into the stomach without recognising the presence of any obstruction. For the eight days he remained in Cincinnati nothing further was done for his relief. In the meantime, on account of pain, the patient took morphia and sustained himself on soft foods and liquids.

Ten days after the injury he went to Indianapolis to attend to professional business, remained there one day, then to Memphis, thence to Atlanta, returning to Cincinnati several weeks after the accident. He then came to New York to consult Prof. R. C. Myles. The latter had a radiograph made which showed distinctly the outline of the plate lying just behind the upper portion of the manubrium, the convexity of its curve looking upward and toward the patient's left shoulder.

Instrumental examination confirmed the location of the foreign body, which so completely closed the esophagus that the finest bougie would not pass it without using a degree of force which was considered unjustifiable. The patient by this time was much emaciated from six weeks of semi-starvation, for the last four days of which he had swallowed only a small quantity of liquids.

1. Read before a special meeting of the Clinical Society of the New York Polyclinic Medical School and Hospital.

*Operation.*—I saw the patient, Sunday, April 9, six weeks after the accident. Dr. Myles made several attempts to remove the obstruction by seizing it with esophageal forceps. It was, however, so firmly imbedded that it could not be dislodged. The patient was removed to the private pavilion of the Polyclinic Hospital, and at 3 o'clock on that afternoon, assisted by Dr. Myles and the polyclinic staff, esophagotomy was performed. Chloroform was selected as the anesthetic, as it was desirable to avoid vomiting during or after the operation.

An incision five and one-half inches in length was made along the anterior border of the left sterno-mastoid muscle, the incision extending one and one-half inches on to the surface of the manubrium. The fibers of the sternohyoid and the sternothyroid muscles were separated by dull dissection, the left lobe of the thyroid body turned upward, and careful traction made on this in order to avoid overtension of the recurrent laryngeal nerve, which was clearly seen as it came from behind the carotid artery and passed upward and inward in the general direction of the inferior thyroid vessel of that side.

In the exposure of the esophagus at this low level very considerable care was necessary to avoid injury to this nerve. A bougie was carried into the esophagus by Dr. Myles, and on this as a guide I made an incision three-quarters of an inch in length through the gullet on its left posterior aspect. This was gradually dilated by inserting the index finger which, carried down to its full length, barely came in contact with the upper rim of the plate. On the finger as a guide a pair of dull-pointed scissors, curved on the flat, was passed anterior to the plate and slightly beyond it. With this on one side and the finger opposing it, the upper corner of the plate, which was deeply imbedded in the walls of the esophagus, was carefully loosened and tilted upward, and in that way dislodged and brought up lengthways to the wound and extracted without any further difficulty. The wound in the esophagus was left open, while the upper portion of the superficial wound was closed with four or five silk-worm gut sutures. The deeper wound was filled with a light packing of sterile gauze. There was no vomiting during or after the operation.

*After Treatment.*—The patient was placed in bed on the back, the foot of the bed elevated about twelve inches in order to facilitate drainage upward and away from the mediastinum. Every four hours he received six ounces of normal salt solution by the rectum for the first twenty-four hours. After this the quantity was increased to eight ounces, and in the intervals two nutritive enemata were given. He suffered practically no pain after the operation.

Nothing passed into the esophagus for the four days from Sunday until Thursday, April 13. On this date a tube was easily introduced through the mouth and esophagus and sixteen ounces of milk were carried into the stomach. This was repeated on the next day. On the following day, the 15th, six days after the operation, he began to swallow liquids, and of sixteen ounces of milk given four ounces came out through the wound. The proportion which leaked through gradually diminished and by April 20, twelve days after the operation, the esophageal wound was entirely healed, and he is now entirely recovered.

### COLONIC ALIMENTATION.

The value of alimentation by the colon as a feature of surgical practice is so important that the following is submitted:

For conveying water by the lower bowel to the blood, normal salt solution should be employed (one teaspoonful of salt to one pint of water which has been boiled and allowed to cool down to the temperature of the body). From six to eight ounces to as much as a pint, and at times more than this, may be employed if absolutely necessary. The foot of the bed should be well elevated so that the liquid will gravitate away from the rectum and thus avoid pressure which induces an effort at evacuation. When there is great intolerance of the lower bowel smaller quantities should be employed with more frequent repetition.

### MILK PREPARED BY THE WARM PROCESS.

One of the most useful foods given by the colon is milk. When the bowel is irritable the warm process, which is as follows, should be employed: Put a teacupful (gill) of cold water and the powder contained in one of the peptonising tubes[1] into a clean quart bottle and shake thoroughly; add a pint of cold fresh milk and shake again; then place the bottle in a pail or kettle of warm water—about 115° F.—or not too hot to immerse the whole hand in without discomfort. Keep the bottle in the water bath for from thirty to forty minutes or longer if a greater degree of pre-digestion seems necessary, then put it immediately on ice. As a portion is needed, shake the bottle, pour out the quantity—usually four ounces—and heat gently to blood warmth. Avoid hasty heating and overheating.

Six, eight, or twelve ounces may be given every four or five hours, or a larger quantity if the case is urgently in need of nutrition and the bowel is tolerant. In rectal feeding it is of great importance not to overcrowd the colon sufficiently to produce irritation.

### COLD PROCESS.

In cases where the enema can be retained for some time without irritation, the milk may be peptonised by the cold process.

1. I prefer those prepared by Fairchild Brothers & Foster.

Put a teacupful (gill) of cold water into a clean quart bottle and dissolve it by shaking thoroughly the powder contained in one of the peptonising tubes; add a pint of cold fresh milk, shake the bottle again and immediately place it directly in contact with the ice.

Warm each portion as it is required for injection, being careful to avoid hasty heating or overheating.

Or, only a sufficient quantity to use may be prepared each time by the following method: In two tablespoonsful (one ounce) of cold water dissolve one-quarter of the contents of a peptonising tube; add eight tablespoonsful (four ounces) of cold milk; warm to the proper temperature and inject at once.

### EGGS.

In administering eggs the following formula is advisable: Dissolve the white of an egg in three times its bulk of warm water; to this add the contents of one of the peptonising tubes, stir well, and inject at once. The water should be just warm enough to make the mixture the proper temperature for the injection, not hot enough to coagulate the albumin.

Another method of using the whole of the egg is to beat the white and the yellow well together, with a pint of milk, adding a gill of water in which the contents of a peptonising tube has been dissolved. It may be employed cold, but if it is thought best to use the warm method, warm water can be used as in the formula immediately preceding.

### BEEF JUICE.

In using beef for rectal feeding, to a tablespoonful of minced lean beef add four tablespoonful of cold water, and gradually heat to boiling. Strain all through a fine sieve or colander. When it becomes lukewarm add the contents of one of the peptonising tubes, and it is ready for injection. More water may be added should it seem desirable.

### PEPTONISED FOOD AND WHEY.

I have used Fairchild's panopepton in rectal feeding with much satisfaction. It should be diluted in two or three parts of lukewarm water, or preferably, normal salt solution.

If it be desired to employ whey, it can be combined with panopepton at times as follows: Put one pint of cold fresh milk in a clean saucepan and heat it to not over 100° F. Add two tablespoonsful of essence of pepsin and stir just enough to mix. Let it stand until firmly jellied, then beat with a fork until it is finely divided, and strain. Warm to the proper temperature and inject without dilution. Panopepton and whey may be used in conjunction by adding three parts of whey to one of panopepton.

Panopepton and peptonised milk may be used by mixing one tablespoonful of panopepton with two or three of peptonised milk prepared by the warm process. Mix the panopepton and peptonised milk when required for use.

